# The “secret sauce” for a mentored training program: qualitative perspectives of trainees in implementation research for cancer control

**DOI:** 10.1186/s12909-020-02153-x

**Published:** 2020-07-28

**Authors:** Rebekah R. Jacob, Angeline Gacad, Christine Pfund, Margaret Padek, David A. Chambers, Jon F. Kerner, Anne Sales, Maureen Dobbins, Shiriki Kumanyika, Ross C. Brownson

**Affiliations:** 1 Prevention Research Center, Brown School, Washington University in St. Louis, 1 Brookings Drive, St. Louis, MO 63130 USA; 2 Center for the Improvement of Mentored Experiences in Research, Wisconsin Center for Education Research, Institute for Clinical and Translational Research, School of Medicine and Public Health, University of Wisconsin-Madison, 1025 W. Johnson St., Madison, WI 53705 USA; 3 Division of Cancer Control and Population Sciences, National Cancer Institute, 9609 Medical Center Drive, Bethesda, MD 20892 USA; 4 Canadian Partnership Against Cancer, 145 King Street West, Toronto, ON M5H 1J8 Canada; 5 Center for Clinical Management Research, VA Ann Arbor Healthcare System, 2215 Fuller Road, Mail Stop 152, Ann Arbor, MI 48105 USA; 6 Department of Learning Health Sciences, University of Michigan Medical School, 1111 E. Catherine Street, Ann Arbor, MI 48109 USA; 7 School of Nursing, National Collaborating Centre for Methods and Tools, McMaster University, 175 Longwood Road South, Hamilton, ON L8P 0A Canada; 8 Department of Community Health and Prevention, Drexel University Dornsife School of Public Health, 3215 Market Street, Philadelphia, PA 19104 USA; 9 Department of Surgery (Division of Public Health Sciences) and Alvin J. Siteman Cancer Center, Washington University School of Medicine, Washington University in St. Louis, 660 S. Euclid Ave. Campus Box 8100, St. Louis, MO 63110 USA

**Keywords:** Mentoring, Mentored training, Dissemination and implementation research

## Abstract

**Background:**

Mentored training approaches help build capacity for research through mentoring networks and skill building activities. Capacity for dissemination and implementation (D&I) research in cancer is needed and mentored training programs have been developed. Evaluation of mentored training programs through quantitative approaches often provides us with information on “what” improved for participants. Qualitative approaches provide a deeper understanding of “how” programs work best.

**Methods:**

Qualitative interviews were conducted with 21 fellows of the National Cancer Institute-funded Mentored Training for Dissemination and Implementation in Cancer to gain understanding of their experiences with mentoring received during the program. Fellows were selected from all 55 trained participants based upon their gain in D&I research skills (highest and lowest) and number of collaborative connections in the program network (highest and lowest) reported in previous quantitative surveys. Phone interviews were recorded with permission, transcribed verbatim, and de-identified for analysis. Codes were developed a priori to reflect interview guide concepts followed by further development and iterative coding of three common themes that emerged: 1) program and mentoring structure, 2) importance of mentor attributes, and 3) enhanced capacity: credentials, confidence, credibility and connections.

**Results:**

Interviews provided valuable information about program components that worked best and impacts attributed to participation in the program. Fellows reported that regular monthly check-in calls with mentors helped to keep their research moving forward and that group mentoring structures aided in their learning of basic D&I research concepts and their application. Accessible, responsive, and knowledgeable mentors were commonly mentioned by fellows as a key to their success in the program. Fellows mentioned various forms of impact that they attributed to their participation in the program including gaining credibility in the field, a network of peers and experts, and career developments (e.g., collaborative publications and grant funding).

**Conclusions:**

These findings suggest that mentored training works best when mentoring is structured and coupled with applied learning and when respected and dedicated mentors are on board. Increased scientific collaborations and credibility within a recognized network are important trainee experiences that should be considered when designing, implementing, and sustaining mentored training programs.

## Background

Years (and lives) are lost between development of evidence-based practice and regular implementation for cancer prevention and control [1]. Dissemination and Implementation (D&I) in cancer research seeks solutions to closing this gap [2]. Training needs for D&I have been recognized and have led to the development of specific educational competencies [3–5]. Several established training programs, varying in scope and format, have been offered and evaluated in the last decade [6–13].

Mentored training approaches are used frequently in pre- and post-doctoral training initiatives and have the potential to increase capacity and networking in scientific fields [14–17]. Previous studies document successful mentoring relationships to be dependent on several characteristics such as clear expectations (from mentors and mentees), shared values, and reciprocity [15, 18, 19]. Trainees of mentored programs previously reported increased scientific productivity and increased skill in D&I research competencies [20, 21], but exactly how and why the mentored component and/or the mentoring network relates to these outcomes remains less clear.

The National Cancer Institute’s R25 Mentored Training in Dissemination and Implementation Research for Cancer (MT-DIRC) began in 2014 with the goal of building capacity for D&I research by providing training and longitudinal evidence-informed mentoring to cancer researchers [21]. The goal of this qualitative study was to expand our program’s evaluation by understanding fellows’ experiences within our uniquely designed mentored training program. Specifically, our main research questions were 1) what aspects of the mentoring program were most beneficial and 2) what role did mentoring play in the fellows’ overall experience of the training program.

### Training program conceptual framework

**Fig. 1:**
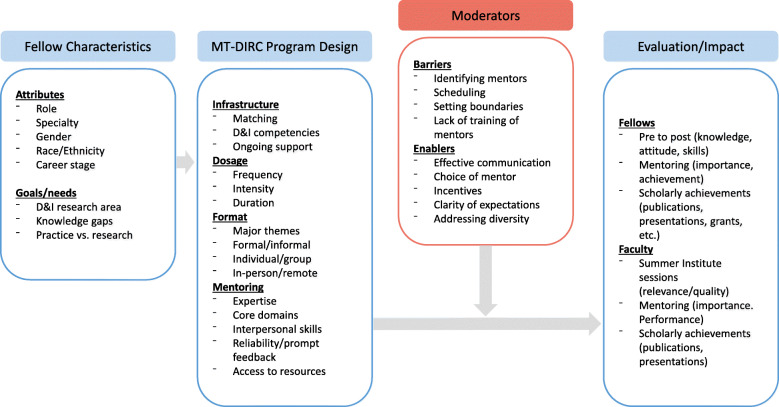
Conceptual model of the Mentored Training for Dissemination and Implementation Research in Cancer program

Our D&I training program was based on educational and social learning theories [15], along with principles of evidence-informed mentoring. Previous research suggests that professionals should be active contributors in the educational process so that learning is work-situated, shaped by their knowledge and experience, and teachers and facilitators should advance learning by providing guidance, support, and constructive feedback [22–25]. The conceptual framework for MT-DIRC was derived from the work on knowledge translation from Gagliardi and colleagues [15]. The framework contains four major domains (Fig. 1), which helped us in designing MT-DIRC, evaluating our progress, and disseminating our findings: 1) trainee characteristics, including the attributes and goals of the trainees; 2) training program design (e.g., the needed infrastructure, mentor characteristics, webinars); 3) moderators that might influence the success of the training program (e.g., mentor training, effective communication; planning meetings); and 4) a set of evaluation measures (e.g., changes in knowledge/skills, research collaborations on publications and presentations).

### Training program description

**Fig. 2:**
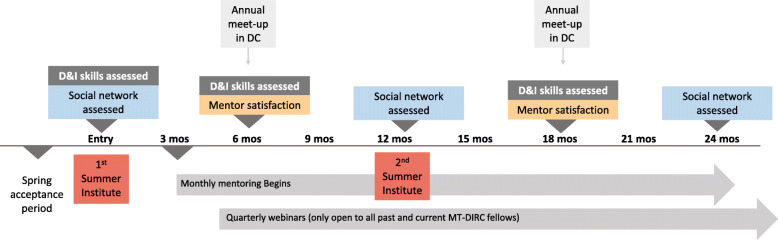
Timeline for fellows of the Mentored Training for Dissemination and Implementation Research in Cancer program

MT-DIRC fellows were selected through a competitive application process. Doctorate-level cancer researchers interested in building their capacity for D&I research were encouraged to apply and submit a research project to work on during the program. Core faculty each reviewed and scored a selection of candidates (three reviewers per application). Allotted fellowship slots (12–14 per year) in the two-year mentored training program were offered to applicants with the highest scores. Figure 2 shows the timeline for a fellow in the MT-DIRC program. Fellows attended a five-day in-person summer training in St. Louis, Missouri in their first and second year. In-person trainings offered didactic sessions featuring core competencies in D&I research [5] along with several hour-blocks of small group mentoring sessions featuring real-time feedback on fellows’ works in progress (e.g. manuscripts, grant proposals, concept papers, data analysis and synthesis).

### Mentoring

Mentor-mentee matches were assigned by aligning research topic areas. Mentors were each assigned 1 to 4 mentees. This number varied from year to year given matriculation of graduating fellows and introduction of incoming fellows. Mentors were senior faculty experienced in D&I research and were identified through previous collaborations, professional networks, and rosters of mentors and faculty from similar D&I training programs [6, 7]. We worked with each mentor’s institution to appropriately navigate monetary incentive (e.g., consultancy pay, % effort) for their contribution to the program. Mentors completed evidence-informed mentor training (synchronous and asynchronous) before mentoring their first cohort of fellows [26–28]. Mentors also attended an informational session at the beginning of each Summer Institute, led by an expert in evidence-informed mentoring. Mentors were encouraged to discuss expectations with mentees with either an informal or formal “mentoring compact” to outline roles and responsibilities for mentors and mentees. Mentors participated in quarterly calls with each other and challenges and successes were shared with all mentors in the program. Mentors in this program were not meant to replace mentoring available to trainees at their current home institution, rather to add an additional layer of mentoring needed to specifically address D&I research needs. Mentors from fellows’ home institutions did not have contact with MT-DIRC mentors.

In addition to the in-person mentoring included in the Summer Institute, fellows also received at a minimum, monthly one-hour mentoring calls either one-on-one or with the other fellows assigned to each mentor. Since each year accepted a new cohort, mentoring groups contained at least one fellow in their second year of the program and one in the first. This was intentional so that newer cohorts could learn from experiences of earlier cohorts.

### Networking

The program featured several components designed specifically to encourage networking. At the Summer Institute, we formed small groups with first year and second year fellows to discuss various aspects of D&I research. Generally, small groups were selected based on area of research focus specified in each fellow’s concept papers, though some years were more diverse than others. In course evaluations of the first two institutes, fellows mentioned that it was helpful to have a second-year fellow give advice on how best to take advantage of the training program. To address this, three alumni fellows were invited to present a special “lessons learned” session at the final Summer Institute to advise fellows on how best to approach the MT-DIRC program to accelerate their work in D&I research. Alumni fellows were also available for one-on-one meetings with fellows and joined small group discussions. We offered two networking dinners at each Summer Institute, one on the first night and one on the night before the last training day to close out the week. Monthly mentoring inherently included networking as groups of fellows and mentors connected on a regular basis for 2 years. A networking dinner for all fellows and alumni fellows was hosted at the Annual Conference on the Science of Dissemination and Implementation in Health, held each year in Washington, DC. We encouraged social media networking with the program’s Twitter account (@mtdirc). Through Twitter, fellows’ accomplishments (presentations, courses, publications, and grant awards) and other program announcements were shared with fellows (and their networks) in addition to the broader implementation science community (#ImpSci). We also shared accomplishments, job announcements, and program announcements by email to all fellows and mentors and posted to the training program’s website (https://sites.wustl.edu/mtdirc/).

### Program evaluation

Fellows completed a baseline (pre-institute) quantitative survey before attending their first Summer Institute. The survey assessed fellows’ perceived competency in 43 D&I research skills and was repeated at 6 months and 18 months in the program. For example, participants self-rated their skill (1 not at all skilled to 5 extremely skilled) in “Describing a range of D&I strategies, models and frameworks,” “Defining what is and what is not D&I research,” and “Identifying common D&I measures & analytic strategies for research.” Satisfaction with mentoring and perceived mentoring competency of each fellows’ assigned mentor was also assessed in the quantitative survey at 6 and 18 months. Mentors were asked to rate their own mentoring competency each December. Additional quantitative survey measures are detailed elsewhere [21]. All fellows and mentors completed a social network survey each June which measured collaborations among fellows and mentors. Finally, qualitative interviews were conducted to understand contextual nuances of the program that were otherwise unavailable through quantitative and network data alone. Quantitative skills and individual-level social network data were used to select participants of the qualitative interviews described in this study.

## Methods

This study used a qualitative descriptive design which allows for a pragmatic approach to examining the experiences of a group of people, sampled purposefully and analyzed with modifiable coding systems [29–31]. For this study, we applied this design primarily to produce a description of common experiences among fellows in the MT-DIRC program to answer the following research questions: 1) what aspects of the mentoring program were most beneficial and 2) what role did mentoring play in the fellows’ overall experience of the training program.

### Participant selection

Skill gain and research collaborations within the program’s network were main outcomes from this study. We hypothesized that those who did not gain as much from the program (skills and research collaborations) may have experienced the program differently from those fellows with the best outcomes. Therefore, we used purposive sampling to include extreme cases in skills and collaborations [32]. Our strategy in selecting participants was, in part, to ensure that we collected experiences from those fellows who faired the best, and those that did not in terms of targeted program outcomes. As described in other publications [33, 34], we examined pre and post skills data to determine skills gained during the program. A skill gain score was calculated by taking the difference of each of the 55 fellows’ overall D&I skills average at time one (pre-institute) from the average at time 3 (18 months in the program). The skill gain score was then divided into tertiles and fellows were selected from the top (highest score or most skills gained) and the lowest (lowest score or least skills gained) tertiles into a pool of potential interview participants. For networking, we examined degree centrality (or the number of collaboration network ties) for each fellow within the June 2018 MT-DIRC network. Specifically, we examined the degree of any collaboration (e.g. research, presentations, grant writing, manuscript writing) reported with other fellows and mentors. Degree was split into tertiles and the higher (highly connected) and the lower (least connected) fellows were selected as potential interview participants. A two-by-two matrix was constructed which included a total of 22 fellows with 1) high D&I skill gain and high network connectivity, 2) high D&I skill gain and low network connectivity, 3) low D&I skill gain and high network connectivity, and 4) low D&I skill gain and low network connectivity. Based on Guest et al. estimates, we determined our sample would be large enough to reach saturation of themes [35].

### Interview guide

Drawing from the program’s overall conceptual model (Fig. 2), we developed a semi-structured interview guide to understand the experiences of fellows who completed the MT-DIRC program. Several of the MT-DIRC mentors, program staff and mentoring expert (CP) provided editing and feedback to the initial interview guide. We then pilot tested a draft interview guide with five alumni fellows of a similar mentored training program for D&I for mental health research [7] who provided additional feedback for clarity. Interview questions were sent ahead of time to each pilot tester and, similar to cognitive interviewing, we asked if the questions were clear and why each was easy or challenging to answer. The final interview guide and questions are presented in Additional file 1. Program staff invited selected fellows via email to be interviewed by phone about their experiences in the MT-DIRC program. Interviews lasted between 21 and 56 min and fellows were offered a $40 Amazon gift card for participating. A Research Assistant, who had not previously interacted with MT-DIRC fellows, conducted the interviews. Of the 22 invited fellows, 21 participated. All interviews were recorded with permission and transcribed verbatim between July and August of 2019 by an outside entity (Rev.com). Human Subjects approval was granted by the Washington University in St. Louis Institutional Review Board (#201903161).

### Coding process

Each transcript was de-identified and uploaded into NVivo [36]. Two of the authors (AG and RRJ) completed all coding. First, codes were developed a priori to reflect each item of the interview guide. The first two transcripts were coded independently and discrepancies discussed to achieve further calibration. Remaining transcripts were coded with further refinement through constant coding comparisons (iterative coding) [37]. The initial codes developed from interview guide questions were as follows: mentoring experience, mentor communication, mentor expectations, D&I skills growth, capacity, networking, career growth, suggested improvements, and scholar advice. Summaries of each code were reviewed separately and thematic commonalities were noted and discussed by the team. Three common themes emerged from initial coding: 1) program and mentoring structure, 2) importance of mentor attributes and 3) enhanced capacity: credentials, confidence, credibility and connections. We then created codes for these three common themes and coded all transcripts with the newly created codes. After coding for common themes, we assessed frequency of the three primary codes across the four sample groups [38].

## Results

### Theme 1: program and mentoring structure

In comparison to their other mentoring experiences, fellows remarked on the value of MT-DIRC’s deliberate and concentrated mentoring approach. Fellows reported mentoring was more focused and targeted around the D&I research they were working on compared to previous mentoring experiences. Fellows also remarked about the value of having structured in-person time for mentoring and devoting time specifically for their D&I research needs and skill building.

*“the ability to have the kind of structured time in person was really critical to being able to really develop skills and develop kind of relationships. So just having in person time. I think the way that the in person time was structured was really well done to have kind of a mixture of the didactic kind of training with more of an in the fishbowl kind of session where you’re bouncing ideas and kind of developing ideas with feedback from your mentor and group. Having the time to be able to actually meet and with the other faculty at MT-DIRC.”*

Fellows mentioned the two-year commitment from mentors or longitudinal mentorship as valuable to build and maintain relationships. In addition, fellows saw the value in attending more than one Summer Institute as they had the added opportunity to discuss lecture material and program readings both with other fellows at the institute and those fellows and mentors in their mentoring group. The opportunity to work one on one with the mentors in person on a specific project was seen as critical to the training. This allowed for feedback in practical application of skills versus “just doing a general training.”

*“the mentoring helped me to gain the skills. The mentoring provided me with role modeling. That includes from [assigned mentor] and the other mentees, so being able to see what other people were doing and learn from their experiences, it also provided me with feedback as I applied the information that we learned from the training and also by providing me with new information as well, so suggestions and specific directions I could follow.”*

The quote above highlights the structure of the multi-layered mentoring network that MT-DIRC designed (primary mentor, near peer mentors, peer mentors, home institutions mentors). Fellows mentioned explicit conversations with their mentors regarding the scope of mentorship and expectations for both mentors and mentees. Mentors and fellows were strongly encouraged to create mentoring plans or contracts. This was generally perceived as useful for fellows, especially initially, to understand what to expect in the next 2 years of the program following the first Summer Institute. For some, expectations were carefully articulated and goals for what the fellow and or group wanted to be accomplish helped in giving structure to what each was working on and how they were going to move forward.

*“setting the expectations at the beginning was really helpful, because, [assigned mentor] was like an unknown person to me. We were unknown to her. We were just kind of put in her group, and we were assigned to her … having explicit conversations about expectations makes a lot of sense almost all the time, but especially in a situation like this.”*

Regularly scheduled mentoring meetings and “homework” given by mentors helped to keep fellows on task with research projects. Setting deadlines, defining deliverables, the “expectation to send progress along” and feelings of being accountable to their assigned mentor was seen as helpful in accelerating work. Having regular meetings on the calendar were instrumental for one fellow in just reminding them of their overall research goals and overall career goals. Some fellows created their own agenda or update document where their progress and updates were shared regularly with the group.

*“It held me accountable to have something to share with the group on a regular basis. It kept me moving. I always had to have a product.”*

Structure of regular meetings to include other peers was also seen as valuable. Fellows learned from other fellows who were in different phases of research or in different academic environments.

*“When someone was really trying to identify what was innovative about their project, in terms of implementation science, I felt that it also applied to me, that I could learn about that, about how to sell my project to an implementation science audience”.*

Fellows also remarked on insights gained from other fellows’ processes in finding funding success in terms of K-awards, R01s and other larger grants.

*“I feel like the team approach was helpful because I was learning a lot from the other two mentees. Well, both mentees had significant funding before. One of them had had a previous R01. And so it was really helpful to hear their processes as well. So I learned a lot, not only from [assigned mentor], but also the other mentees as well.”*

### Theme 2: importance of mentor attributes

In addition to structure, fellows commonly noted the contribution of their assigned mentor a key to an overall positive program experience.

*“I think the faculty are key … .having excellent faculty who are incredibly knowledgeable is key but then faculty who really care about the fellows and really care about the program. I think that’s basically, that’s probably the secret sauce.”*

Fellows had positive experiences with mentors who were accessible, confident and open to alternatives, open to listening and providing feedback, vested in the fellow as an implementation scientist, and focused on career trajectory and the bigger picture and not just day-to-day activities. In addition, fellows appreciated when mentors came with a wide range of career experience. Fellows mentioned having someone outside their institution as a benefit to keep mentoring more focused on D&I research in general (versus day to day work at the institutional level) and also to get an external perspective or neutral angle.

“*… having a mentor who is really just there to support me and kind of take my lead on where I need development and they don’t have as much riding on my success, it’s just that they want to see me do well, I think that distinction is a little bit different then what I’m used to.”*

Diversity of the mentors’ disciplines and academic background was also mentioned by fellows as helpful in learning.

*“I also appreciated the diversity of faculty. People were from different disciplines, different geographic areas. They had different levels of focus in terms of public health research and cancer research specifically … that was really helpful … I’ve been able to look at ideas from other areas and get inspired by those.”*

Having a mentor that reviewed materials in a timely manner was also important to fellows. Having mentors who “have the time and are able to set aside some time to do it” was mentioned by one fellow as the key for a program like MT-DIRC. One fellow specifically mentioned a time when they needed feedback within a short timeframe, and were surprised that their assigned mentor prioritized this need, further demonstrating the mentor’s commitment to the fellow. Fellows expressed gratitude for mentors who made an effort to review information fellows sent ahead of meetings and were “prepared” with “appropriately critical” feedback for fellows. Having experts in the field that were “available” when needed and accessible also aided in receiving help along the process. Fellows noted the “welcoming” environment that the mentors of the program helped to create where it was safe to have questions and challenges.

*“They really respected the fellows. They treated our ideas with respect and were really generous with their time. So the presentations were really good. The mentoring itself was really good, and then the atmosphere, the culture was very collegial and welcoming and respectful.”*

### Theme 3: enhanced capacity: credentials, confidence, credibility and connections

Fellows expressed the mentored training experience as essential for a variety of self-defined successes, or enhancing their capacity or D&I research. Fellows reported participating in the program helped to give them credibility in the field of D&I for cancer research, and demonstrated their expertise gained. With a newer field like D&I research, one fellow stated the benefit of participating was to provide “credentials,” something with which to introduce oneself. Another fellow stated that “there’s no way I would’ve even felt like I could speak up at this conference last week without that behind me.” According to one fellow, the training was the “antidote to imposter syndrome” that they experienced in certain settings. One fellow made the point that senior people in D&I did not have this opportunity or structured learning in D&I, and so, being able to tell others of their membership in a formal training program was a “huge way” to open doors.

Another piece of this credibility was the fellows’ own perceptions of their competence in D&I research skills or as one fellow put it, “starting to see myself as an implementation scientist and feeling confident to have enough of a sense of the field and what I do.” Some fellows mentioned becoming the “go-to” D&I research expert at their institution, reviewing and giving advice on other’s D&I grants.

*“I was able to use this education when I gave back to my institution and I joined our internal pilot grants that we have here. Some are in implementation science, so I was invited to join the review panel for those grants. That helped give me some additional recognition within my institution.”*

Fellows mentioned the program being instrumental in grant writing, especially for D&I research audiences, and obtaining subsequent funding. One fellow stated that they were “inspired to write a grant in a way that [they] probably otherwise wouldn’t have written it.” Another fellow stated the time to work on and the resources available through the program around their project in the program contributed to their project ultimately being funded.

*“Well, honestly, I would say that I don’t know that I would be where I am in terms of working on an R01 application focused on dissemination and implementation science if it wasn’t for the mentoring program because I was able to gain knowledge that I need to work on this proposal. But then I also got feedback and mentoring. And I have a sense of confidence that I’m going in the right direction.”*

Fellows reported the program helped to define research goals and overall transformed or shaped fellows’ careers, writing grants and manuscripts that focused more on implementation science topics for the first time.

One fellow’s success was the establishment of a new D&I research class at their institution which they co-teach with another fellow. The fellow attributed the course to their participation in the MT-DIRC program. Another fellow mentioned that being able to teach others what they had learned was the “best outcome” from participating in the program. In particular, this fellow began a one-day workshop in D&I research prior to a society conference and described it as a “really nice opportunity for us to spread horizons” since the group in attendance included various countries. For one fellow, having a symposium proposal accepted for presentation at the biennial Society for Implementation Research Collaboration meeting was a success attributed to participation in the fellowship. Two fellows collaborated on a session accepted for the Annual Science of D&I conference in DC, which was seen as beneficial from the small group mentoring structure.

The program’s network of fellows and mentors, and the collaboration within it, was commonly mentioned by fellows as resulting in considerable professional impact. This collaborative energy was cited as impetus for writing papers together and for four fellows, recently submitting a pilot grant together. Another fellow reported having their assigned mentor connect them with another MT-DIRC mentor with an implementation-ready intervention, ultimately leading to awarded grant funding. Fellows also reported the program’s network increased their professional reach.

*“It gave me a personal connection with people from all over the country and all over the world in many cases. And we are still in communication on a somewhat regular basis. Once you find these nodes, these MT-DIRC fellows, they have their own network that they can then refer me to.”*

This reach provided a group of people with whom fellows could always “touch base.” Especially at the annual meeting, fellows looked forward to seeing other cohorts of fellows and other mentors. One fellow mentioned networking at conferences was particularly important because of the earlier stage of their career.

Another fellow saw networking as a benefit from the program that will last beyond the 2 years in the fellowship.

*“I can see it being more of a help in the future to be able to cold email somebody and say, ‘Hey, we were in the MT-DIRC program together.’ Or ‘You were [part of my MT-DIRC cohort] faculty. Do you mind, I would love your advice on this.’ Or ‘Here’s a potential thought I had for a collaboration.’”*

### Challenges

Fellows mentioned challenges during their participation in the MT-DIRC program. In general, challenges were often the reverse of or counter to the major themes detailed previously.

A fellow spoke about the challenge of switching assigned mentors a year into the program (or interrupted longitudinal mentoring). Even so, the fellow described the in-person session as the additional layer to the program which made up for the sub-par mentoring received in the first year of the program.

*“I would have been very happy to have monthly meetings as a group hear what people are working on learn about D&I concepts. That just wasn’t what [the mentoring relationship] was … I think that [1st assigned mentor] did very little for me … anything would have been more … the face-to-face sessions for the MT-DIRC program, where we were there for a week in St. Louis were amazing and I took away so much from that, and I met a ton of really important people who likely I’ll reach out to and collaborate with. And so my experience for MT-DIRC was absolutely transformative, but the mentoring part I think was not what I had hoped for. I would say I still got an amazing amount of stuff out of the MT-DIRC program, but the mentoring part I did not.”*

In addition, fellows’ expectations for engagement beyond their assigned mentor (or the mentoring network) were not always met.

*“I talked to a different person who wasn’t my assigned mentor, but one of the faculty, and I brought him an idea I had for a paper. It kind of got off the ground, but then I was never really able to get on his radar screen again, once [the Summer Institute] was over. So that one kind of petered out, and I wish it hadn’t.”*

Finding time to devote to the weeklong training in St. Louis was likely a challenge for many fellows and was mentioned specifically by one fellow with young children.

*“It’s a bit of a tough time of the year to be able to go away for a week. That was always a challenge. We have young kids and I think a lot of the fellows also do. It’s just hard to tear away during graduation and recital season.”*

For a few fellows, the differences in research area for their mentor was seen as a barrier in the mentoring process in their particular research focus.

*“the hard part for me was that [assigned mentor] and I are in very different areas, and I’m kind of in different areas from a lot of the other folks. So I kind of got generic mentoring, not specific to some of my work typically. But given that, it was still useful.”*

Similarly, fellows mentioned having been matched with mentors who operated in different funding systems was challenging in terms of understanding the various requirements of each system.

A few external challenges were mentioned in terms of making progress on research during the program. One fellow mentioned their institute had a fairly well-founded D&I research capacity core, and the challenge was more in terms of getting in with an already formed infrastructure. Conversely, not having formal institutional capacity for conducting and building capacity for D&I research was a barrier for one fellow in terms of developing partnerships.

*“I’m in a very odd kind of situation here at [institution C]. I am the only faculty member in my particular context … I’m not part of an academic department … I am the only faculty member where I am. I don’t have colleagues who are partnering with me on a regular basis.”*

In a different example, one fellow remarked about the unique challenge of providing D&I research consult to others in newer field because “not everybody knows what it is,” which was clear to the fellow because “a lot of the consult requests would not actually be implementation science.”

### Discussion

These interviews provided valuable information about the components of MT-DIRC that were most beneficial to fellows and the role that mentoring played in the fellow’s overall experience of the program. In answering our research questions and evaluating findings against our program’s conceptual model, we confirmed the importance of mentor characteristics and program structure and format in designing and implementing mentored training programs.

Fellows were not specifically asked about what makes for an effective mentor. Instead, fellows commonly responded with features of an effective mentor to several interview guide questions, reiterating the importance of the personality and characteristics of the mentor- a key concept in mentoring literature [18, 39] and specified in our program’s conceptual framework. This also speaks to the importance of training organizers to carefully consider the quality of mentor-mentee matching. Straus et al. organized their qualitative study to delineate the characteristics of an effective mentor and the actions of effective mentors [18]. In our study, actions and attributes were fairly entangled suggesting the interplay between both. For example, confident and knowledgeable mentors with many years in the field may be more likely to “open the door” for fellows through deliberate introductions to others within the mentors’ large network. Likewise, populating mentored training programs with key faculty who have mentorship experience and who are dedicated to providing good mentoring is crucial and can make or break a program’s success and its impact.

Fellows reported the mentoring relationships coupled with the structure of the training program were beneficial for progressing in D&I research. Designing mentored training programs that introduce regular contact with both mentors and other peers may be beneficial for keeping research on track, learning from both mentors and peers, and overall connecting didactic material with application to research. These concepts were emphasized by fellows in all skill gain and social connectivity categories. Regardless of fellows’ skills gained during the program or the amount of their network growth, the mentorship component was seen as important – in its structure, the role of the mentors and the impact. In other words, mentoring may level the playing field across diverse backgrounds and factors.

Scientific productivity in terms of manuscripts and grant funding is often a common measure of “success” in academic training programs. While our fellows reported scientific productivity (especially focused in D&I research), perhaps as important was the mention of credibility in the field of D&I. New found confidence as leaders in a relatively newer research field such as D&I research suggests the value of bidirectional relationships with membership in a recognized mentoring network, thus building skills and scientific self-efficacy. The qualitative inquiry provided unique insights which enhance our previous quantitative findings [21, 33, 34] and add depth to our program’s conceptual framwork, specifically in the impact domain.

Understanding how membership in a network of other program trainees can build capacity within a field is a key finding from our work. In designing mentored training programs, unique ways to network within the program design can be built and perhaps can be branded for national and international recognition. For example, our program’s Twitter handle was used mainly to promote applications to the program and to share accomplishments of the fellows and mentors. In addition, the handle was often included in posts from the National Cancer Institute’s Implementation Science handle (@NCI_ImplSci) for promotion of D&I trainings, webinars and workshops. Twitter usage among academics (#AcademicTwitter) is gaining momentum as a prominent tool for career advancement [40], and has grown in the area of D&I (#ImpSci, #DIScience19) [41]. Because of these usage trends and the active engagement (likes, retweets, follows) this handle received, it is likely that the program’s information gained recognition among academics interested in D&I, further establishing credibility of the fellows.

The findings also point to the importance of designing programs which deliberately involve peer mentoring. Decastro et al. previously highlighted the unique abilities of mentoring networks to address possible diverse mentoring needs of a mentee and the importance of “horizontal mentoring” [14]. Our results suggest that having membership in the network led to additional career impact. This idea is highlighted by Luke and colleagues’ work which showed the likelihood of scientific collaborations after 2 years in a mentored training program were predicted by the number of mentoring connections established near the beginning of the program [42]. That is, more mentoring received in a network (either by a faculty or peer mentor) equates to more collaborations (on publications, grants, teaching), and further capacity building within a field.

Contrary to our hypothesis that drove our purposive sampling, we did not find a clear pattern in differences between mentions of benefits and challenges in the main themes across the fellows’ levels of skill gain and network connections. For example, challenges of competing interests and time were mentioned by fellows with high and low skill gain and with a large and small number of network connections. This finding may highlight latent items unmeasured within the conceptual framework such as intrapersonal receptivity to programs, support from local mentors, or some other key variables in the fellow characteristics domain or other domains. It may also mean that many of the underlying reasons for how mentoring is beneficial are consistent across a diverse set of mentees.

### Future directions

The lessons learned from these qualitative evaluation findings will be integrated into the recently funded R25 Institute for Implementation Science Scholars (IS-2) [43] which focuses on applying D&I research to eliminate chronic disease disparities. Further research might also extend to additionally include the program’s mentors in qualitative interviews since mentorship is bidirectional. There were few (*N* = 6) international fellows, though none were represented in these interviews. Future evaluations of mentored training programs should include perspectives from multiple countries, especially when mentors are not in the same country as the fellow and the local context may differ. Some of the challenges to date with outcomes from mentored training programs like ours are small sample sizes and limited years of funding which further limit the ability to combine samples over time. Validated evaluation measures across similar training programs would make cross-project research more feasible when examining quantitative relationships within a conceptual framework.

## Conclusions

Results from these qualitative interviews reveal the “secret sauce” of the mentored training program which contributed to the fellows’ success. Key ingredients include mentoring that is structured and coupled with applied learning and respected and dedicated mentors. These can be built into programs more explicitly (e.g., evidence-informed mentoring, mentoring contracts, structured regular communication). Increased scientific collaborations and credibility within a recognized network were important trainee experiences. This enhanced capacity may require additional contextual understanding to deliberately build into mentored training programs.

## Supplementary information

**Additional file 1.** Final interview guide. This file provides the final interview guide used with fellows of the MT-DIRC program to complete the qualitative interviews described in this study.

## Data Availability

Additional qualitative data analyzed during the current study, which is not published in this article, are not publicly available due to participant confidentiality but may be made available from the corresponding author on reasonable request.

## Abbreviations

D&I: Dissemination and Implementation
MT-DIRC: Mentored Training for Dissemination and Implementation Research in Cancer
