# Supplementary material for: The “secret sauce” for a mentored training program: qualitative perspectives of trainees in implementation research for cancer control
Source: BMC Med Educ. 2020 Jul 28;20:237. doi: 10.1186/s12909-020-02153-x (PMC7385963; doi:10.1186/s12909-020-02153-x)
Supplement: Supplementary file 1 — Additional file 1. Final interview guide. This file provides the final interview guide used with fellows of the MT-DIRC program to complete the qualitative interviews described in this study. [file 12909_2020_2153_MOESM1_ESM.pdf]

INTRODUCTION

Project: Mentored Training for Dissemination and Implementation in Cancer (MT-DIRC)

Hello. My name is _______________. I am from the Prevention Research Center at Washington University in St. Louis and I am calling regarding the Mentored Training for Dissemination and Implementation in Cancer (MT-DIRC) program for which you were previously a Fellow.

We would like to interview you regarding your thoughts on mentoring relationships in general and more specifically, the mentoring you received as an MT-DIRC fellow. This includes facilitators, and barriers for successful mentoring. This interview should take about 45-60 minutes. You are able to stop the interview at any time. Does this time still work for you?

[YES: continue reading below]

[No: Is there a better time we could schedule this interview for to best fit your schedule?]

With your permission, we would like to audio record our conversation for note-taking purposes. Any information we gather will only be released as aggregated, group-level information and will not be attributed to any individual. We will gladly share what we learn with you.

You also have the option to receive a $40 amazon gift card in appreciation of your time.

You may skip any questions you do not feel comfortable answering at any time.

Would you like to proceed with this interview?

[YES: continue reading below]

[NO: Well thank you for your time and consideration about this project. We will not contact you further about participating in these interviews**. END THE PHONE CALL].**

Any questions before we get started?

MT-DIRC MENTORING QUESTIONS

To start off, we're going to ask you a few questions about the mentoring you have received as a fellow in the MT-DIRC program. For each question, we will ask you to specify the mentoring source to which you are referring. For example, [NAME] was your assigned mentor in the MT-DIRC program, core faculty are the other faculty assigned to mentor other MT-DIRC fellows, and guest faculty are any faculty that were guest presenters in the summer institute. These questions are not about mentors at your home institution.

1. To begin, we'd like you to please tell us about your general mentoring experience in the MT-DIRC program compared with other mentoring experience you've had so far in your career (e.g. doctoral, post-doctoral, early career).

2. Please tell us a little bit about the format of your monthly mentoring calls with your assigned mentor. For example, were they with a team, scheduled regularly, were materials sent ahead?

What was helpful? What was not so helpful?

Is there anything else you would have liked your mentor to do? Discuss with you?

3. What specific topics were most often covered in your communication (either in person or virtually/phone calls) with your assigned mentor?

4. During your time in the MT-DIRC program, did you have a discussion with your assigned mentor that explicitly addressed mentor/mentee expectations? Some examples may include an informal or formal mentoring contract or outlining roles, responsibilities and deliverables with timelines.

If so, was this helpful? Why or why not?

If not, do you think this would have been helpful? Why or why not?

5. Is there anything you personally would have done differently in terms of taking part in the mentoring relationship or taking advantage of the mentoring that was available?

6. Is there anything else about your MT-DIRC mentoring we haven't talked about yet that was helpful? Or that could be improved? When answering, please specify who served the mentoring role you are describing either your assigned MT-DIRC mentor, other faculty, guest faculty, other sources.

D&I skill and capacity building

You may remember that one of the goals of MT-DIRC was to build capacity for D&I research through skill building. The in-person Summer Institute sessions at MT-DIRC played a large role in this, but we'd like to hear a little bit about how the mentoring component of the program may have played a role in your skill building.

7. Are there specific skills that mentoring you received in MT-DIRC has helped you in building?

Some examples might include defining what is and what is not D&I research, describing a range of D&I strategies, models and frameworks, and identifying common D&I measures.

Also, this could have included more general research skills like grant writing and writing for publication.

8. Do you think the mentoring you received in MT-DIRC helped you to gain these skills? If so, how?

For example, thinking about your D&I skills level before starting in the MT-DIRC program to now, how do you think the mentoring component of the program facilitated or complemented any gains in skills? Consider any specific conversations you may have had with a mentor that helped your skill building or a resource shared by a mentor that was useful.

9. Has your involvement in MT-DIRC helped you to build capacity for D&I at your own institution? If so, how?

Examples might include designing an "intro to D&I science" workshop, providing consultation, or leading a graduate course focused on D&I.

MT-DIRC NETWORKING QUESTIONS

Now we are going to talk specifically about your networking (with other peers, mentors, guest faculty, and beyond) during your time in MT-DIRC.

10. Did your involvement in MT-DIRC contribute to your networking and connections with others to further your D&I research? If so, how?

11. Do you feel that the MT-DIRC network contributed to your success? If so, how?

12. Tell us about both the value of networking with MT-DIRC faculty and the value of networking with other MT-DIRC fellows? How were these the same or different?

13. Since you began in MT-DIRC, have you informally mentored or advised other MT-DIRC fellows or have you received informal mentoring or advice from other MT-DIRC fellows. If yes, was it helpful? How?

WRAP-UP QUESTIONS

We have just a few more questions to ask before we close.

14. Overall, do you feel that the training, mentoring, and networking provided through MT-DIRC helped your growth as a D&I scientist? If so, how? What else is needed (either from a mentored training program or other support)?

15. What advice would you give to a person/institution who wants to replicate a program like MT-DIRC? Any advice on improvements?

16. If you could give input to junior scholars who are beginning their careers in D&I science, what would be your single most important piece of advice related to mentoring?

17. Is there anything else you'd like to share with us about the MT-DIRC program or MT-DIRC mentoring?

[END]

That is all the questions we have for you today.

Do you have any questions for us before we end?

Thank you for taking the time to speak with us. We will share findings with you as they become available.
